# Design of CGMP Production of ^18^F- and ^68^Ga-Radiopharmaceuticals

**DOI:** 10.1155/2014/680195

**Published:** 2014-09-02

**Authors:** Yen-Ting Chi, Pei-Chun Chu, Hao-Yu Chao, Wei-Chen Shieh, Chuck C. Chen

**Affiliations:** PET Pharm Biotech Company Limited, 1-2F No. 143 and 1-2F No. 141 HouGang, Xinzhuang District, New Taipei City 24257, Taiwan

## Abstract

*Objective.* Radiopharmaceutical production process must adhere to current good manufacturing process (CGMP) compliance to ensure the quality of precursor, prodrug (active pharmaceutical ingredient, API), and the final drug product that meet acceptance criteria. We aimed to develop an automated system for production of CGMP grade of PET radiopharmaceuticals. *Methods.* The hardware and software of the automated synthesizer that fit in the hot cell under cGMP requirement were developed. Examples of production yield and purity for ^68^Ga-DOTATATE and ^18^F-FDG at CGMP facility were optimized. Analytical assays and acceptance criteria for cGMP grade of ^68^Ga-DOTATATE and ^18^F-FDG were established. *Results.* CGMP facility for the production of PET radiopharmaceuticals has been established. Radio-TLC and HPLC analyses of ^68^Ga-DOTATATE and ^18^F-FDG showed that the radiochemical purity was 92% and 96%, respectively. The products were sterile and pyrogenic-free. *Conclusion.* CGMP compliance of radiopharmaceuticals has been reviewed. ^68^Ga-DOTATATE and ^18^F-FDG were synthesized with high radiochemical yield under CGMP process.

## 1. Introduction

### 1.1. Trends in Radiopharmaceutical Development

Molecular imaging agents play a major role in drug discovery and development because of their ability to quantify drug properties* in vivo*. Among molecular imaging modalities, positron emission tomography (PET) imaging agents are the most sensitive and could provide target specific information. PET agents show high specific activities since they are made through a nuclear transformation and used carrier free forms of isotopes. PET agents do not produce detectable pharmacologic effects but provide important information concerning the characterization of various diseases. PET agents are able to assist in the determination of optimal therapeutic dosing, delineate differential diagnosis between responder and nonresponder, and predict treatment response by selecting patient who may respond to therapy.

There are two categories in PET radiochemistry. The first category is known as covalent chemistry using cyclotron-produced organic isotopes such as ^18^F or ^124^I to incorporate into molecules. For instance, ^18^F-fluorodeoxyglucose (FDG), a gold standard for PET, has been successfully used to image diseases with high glycolysis. However, FDG has several limitations that give rise to false positive/negative diagnosis and poor predictive value of chemoradiation therapy to tumor response [1]. In addition, certain tumors such as neuroendocrine type tumors have poor uptake of FDG. Therefore, there is a high demand to develop new radiopharmaceuticals beyond FDG in oncology field. In contrast with cyclotron-produced isotopes, the second category employs coordination chemistry using metallic radionuclides produced from generators. A generator uses a parent-daughter nuclide pair wherein a relatively long-lived parent isotope decays to a short-lived daughter isotope for imaging. The parent isotope, which is produced at a cyclotron facility, can be shipped to a clinical site and is the source from which the daughter isotope is readily eluted. ^68^Ga-based (68-minute half-life, *β* ^+^ = 89% and EC = 11%) PET agents are with significant commercial potential because the isotope can be produced from a ^68^Ge/^68^Ga generator (275-day half-life or 18-month shelf life) on site and is a convenient alternative to cyclotron-produced PET isotopes, such as ^18^F or ^124^I. The short half-life of ^68^Ga permits applications with suitable radioactivity while maintaining patient dose to an acceptable level. Furthermore, ^68^Ga^3+^ can form stable complexes with many ligands containing oxygen and nitrogen as donor atoms. This makes ^68^Ga suitable for chelation in various molecules. Over the last three decades, several ^68^Ge/^68^Ga generators have been proposed in an attempt to provide high yield of ^68^Ga and low breakthrough of ^68^Ge. The efforts in image-guided therapy, theranostic (image and therapy) approaches, pivotal clinic trials, and integration with system biology findings on genomic and proteomic expressions are the trends for PET agent development in patient care management.

### 1.2. Need for Automated Synthesis of PET Radiopharmaceuticals

Radiopharmaceutical chemistry requires intricate handling of radioactive materials, fast reaction times, ease of synthesis, and reproducible results. In the preclinical setting, radiopharmaceuticals are typically synthesized manually. Such applications use* in vitro* and small animal models to validate the agent and require low levels of radioactivity. The use of manual synthesis for clinical imaging, however, is challenging for multiple reasons: (1) clinical agents must meet strict sterility and pyrogenicity requirements which are validated from batch to batch; (2) batch-to-batch reproducibility is required to demonstrate suitable radiochemical yield, radiochemical purity, and other quality control analyses; (3) synthesis time must be fast when dealing with radionuclides with a short half-life; (4) clinical studies require multiple patient doses and would expose radiochemists to much higher levels of radioactivity; and (5) production cost and availability of the technology may limit the viability of the agent in routine clinical practice. The Food and Drug Administration (FDA) permits radiopharmaceuticals produced under well-controlled conditions in central commercial facilities to be distributed to local clinics where they are administered. In addition, radionuclide generator systems produced in well-controlled facilities have gained FDA acceptance and have a long history of successful clinical application. The competitive advantage of generator-based agents lies in their convenient synthetic schemes; however, this attribute is greatly diminished if the tracers lack clinical usefulness. An automated apparatus is needed to assure production efficiency and minimize the radiation exposure by PET isotopes. Regardless of classification, all medical devices are subject to general controls and baseline requirements of the Food, Drug and Cosmetic (FD&C) Act as well as the provisions and standards contained in the USP General Chapter 〈823〉* Radiopharmaceuticals for Positron Emission Tomography-Compounding *and General Chapter 〈1015〉* Automated Radiochemical Synthesis Apparatus*. It is, therefore, critical to consider these guidelines while designing the building blocks of the automated apparatus.

### 1.3. Current Good Manufacturing Practice (CGMP) Compliance in Automated Radiopharmaceutical Production

Radiopharmaceutical production process must adhere to CGMP compliance to ensure the quality of precursor, prodrug (active pharmaceutical ingredient, API), and the final drug product that meet acceptance criteria. The FDA Modernization Act of 1977 (Section 121) requires the FDA to establish approval procedures and cGMP for PET drugs. In 2002, the FDA published a guideline called “Chemistry, Manufacturing, and Controls” dealing with three common PET drug products (i.e., ammonia ^13^N, ^18^F-FDG, and sodium fluoride ^18^F injections) [2]. Even though these guidelines do not apply directly to ^68^Ga, they play important roles in our design review. The FDA has released the CGMP requirements (to be CFR 21 Part 212) and guidance documents for PET drug products on September 15, 2005. The cGMP regulations are the minimum set of requirements for a facility to develop commercial use agents and will be complied with during the commercialization of the automated synthesizer as they apply. The following documents, as outlined in the cGMP guidance, are relevant for putting together the design specifications, installation, verification, and maintenance protocols for the automated synthesizer.

- FDA.* Part 210—Current Good Manufacturing Practice in Manufacturing, Processing, packing or Holding of Drugs*. August 1996.
- FDA.* Part 211*—*Current Good Manufacturing Practice for Finished Pharmaceuticals*. August 1996.
- FDA.* Guide to Inspection of Computerized Systems in Drug Processing*. February 1983.
- FDA.* General Principles of Process Validation*. May 1987.
- FDA.* Sterile Drug Products Produced by Aseptic Processing*. June 1987.
- FDA. 21 CFR Part 11; Electronic Records; Electronic Signatures. FR Notice 7/21/99 (64 FR 39146).
- U.S. Pharmacopeia. 〈823〉* Radiopharmaceuticals for Positron Emission Tomography-Compounding*. USP 26, NF 21, 2003.
- U.S. Pharmacopeia. 〈1015〉* Automated Radiochemical Synthesis Apparatus*. USP 26, NF 21, 2003.

The specific steps for quality assurance and validation are specified in these documents.

- U.S. Pharmacopeia. 〈1〉* Injections*. USP 26, NF 21, 2003.
- U.S. Pharmacopeia. 〈71〉* Sterility Tests*. USP 26, NF 21, 2003.
- U.S. Pharmacopeia. 〈85〉* Bacterial Endotoxins Test*. USP 26, NF 21, 2003.
- U.S. Pharmacopeia. 〈621〉* Chromatography*. USP 26, NF 21, 2003.
- U.S. Pharmacopeia. 〈821〉* Radioactivity*. USP 26, NF 21, 2003.

The CGMP compliance covers manufacturing process and facility, quality guidelines, and personnel training. The Pharmaceutical Inspection Convention and Pharmaceutical Inspection Co-operation Scheme (jointly referred to as PIC/S) are two international instruments between countries and pharmaceutical inspection authorities, which provide together an active and constructive cooperation in the field of Good Manufacturing Practice (GMP). PIC/S's mission is to lead the international development, implementation, and maintenance of harmonized GMP standards and quality systems of inspectorates in the field of medicinal products (http://picscheme.org/). At PET Pharm Biotech firm, we have adopted PIC/S guideline and USP 797 guidance for the production, formulation, and dispensing of PET radiopharmaceuticals. Current regulations and quality standards including FDA regulations and CFR, EU-GMP guidelines, WHO-GMP guidelines, and roles of PIC/S in international regulatory affairs are well documented in online resources. Our team has designed, built, and validated an automated bench-top system for PET radiopharmaceuticals. Here, we report two agents: ^18^F-FDG and ^68^Ga-DOTATATE. The guidance documents were from the FDA relevant CFR 21 part 212, “Current Good Manufacturing Practices for Positron Emission Tomography Drug Products” Final Guidance for Industry and FDA Staff, Center for Devices and Radiological Health, March 2002. We adhere to FDA and PIC/S guidelines for the synthesis of radiopharmaceuticals included in the USP 797 to ensure that the products can be produced using our systemunder the required conditions.

### 1.4. Materials and Methods

**Figure 1:**
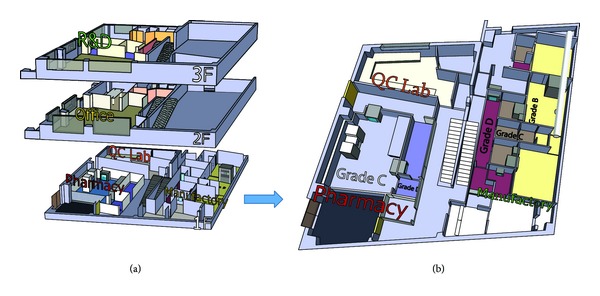
Design of a CGMP facility ((a) a three-floor design containing CGMP process at level 1, data management at level 2, and research development at level 3; (b) an expanded diagram of CGMP facility) at PET Pharm Biotech.

Entered via a series of small rooms increasing in the cleanliness grades, a grade B final gowning room opens to two grade B production rooms (Figures 1 and 4). In each production room (12 m^2^), stands one custom-made hot cell (BqSv, Inc., Taiwan) with two main compartments. The synthesis compartment is maintained in the same level of cleanliness as the room while the aseptic filling compartment is a grade A isolator. Inside this grade A isolator, the particle counts are monitored continuously during filling of the final product by a semiautomatic apparatus and inlet is installed for hydrogen peroxide disinfection.

### 1.5. Key System Features during the Design Phase for ^68^Ga-Agent

- *Turnkey* solution to fully automate the process including syringe drives to elute generator and inject reagents into the vessels, N_2_ gas regulator, and vacuum pump.
- *Purification* of ^68^Ga by eluting ^68^Ga from the generator before synthesis to produce clinical-grade ^68^Ga using a purification cassette, a vial for collection and concentration of peak fraction of elution, an ion exchange column to trap ^68^Ga, and a vial for waste collection of ^68^Ge monitored by a collimated radiation detector.
- *Formulation* of ^68^Ga conjugation with precursor and proper dilution for injection: this panel will have a disposable cassette, a smart reaction vessel receiving the clinical-grade ^68^Ga from the purification panel, and an infrared heater.
- *Disposable cassettes* for 100% tubing replacement to eliminate possible contamination between runs.
- *Automated dispensing* of reagents and solution* via* programmable syringe drives.
- *Real-time monitoring* of sample preparation and radioactivity at several points of the system using a real-time display.
- *Breakthrough monitoring* of ^68^Ge (parent of ^68^Ga) at the purification panel.
- *Data recording* of all experimental data (including pressure, temperature, mass flow, reagents nature, and quantities) in tab delimited ASCII format that is readable in Microsoft Excel. Also, recording of the elution profile of the ^68^Ga generator is desired.

### 1.6. Development of the Hardware and Mechanical to Fit in Hot Cell

This task includes the detailed mechanical design of the Scintomics module basic system. This activity was composed of the following tasks.

1. Develop the overall mechanical system layout.
2. Identify the mechanical subassemblies and produce drawings of the resulting high-level mechanical architecture.
3. Conduct a review of the high-level mechanical architecture.
4. Complete the detailed design of the mechanical subsystems.
5. Produce detailed mechanical drawings and bills-of-material.
6. Identify critical and long lead-time components.
7. Place orders for individual parts, commercially available subassemblies, and vendor fabricated items.
8. Mechanically assemble the basic system instrument module.
9. Verify the mechanical assembly.

### 1.7. Development of the Hardware: Electronics

This activity includes the design of any electronics needed to support the operation of any sensors, motors, or other electronic devices used in the implementation of the basic system instrument module. This activity was composed of the following tasks.

1. Develop the high-level architecture of the Scintomics module system electronics.
2. Identify devices needing the design of custom interface circuitry.
3. Conduct an internal review of the high-level electronics architecture.
4. Complete the detailed design of all electronic subsystems.
5. Produce circuit diagrams, complete the design of any necessary custom PCB's, and produce the corresponding bills-of-material.
6. Identify critical and long lead-time items.
7. Place orders for individual parts, PCB's, and off-the-shelf components.
8. Assemble the module electronics and perform preliminary troubleshooting.
9. Install the module electronics into the module's mechanical assembly.

### 1.8. Development of QA Tests for PET FDG or ^68^Ga-DOTATATE Measurements

Prior to clinic studies, we also conduct testing for the membrane filter integrity, pH, sterility, and pyrogenicity of drug product. The QA criteria are described below.

- *Appearance*: Solution must be colorless and free from particulate matter. The solution inside the collection vial before final release into the collection vial can be recorded by the Web-Cam. Lighting conditions must be consistent and optimal for this test. Test must be completed before release of the drug product.
- *Radiochemical purity/yield*: Radiochemical purity was assessed by HPLC and radio-TLC. The TLC chromatogram (silica gel coated plates) was scanned for distribution of radioactivity in a radio-TLC scanner. Using saline as the mobile phase, ^68^Ga-DOTA migrates near the solvent front, while ^68^Ga-DOTATATE remains at the origin. The retardation factor (*R*_*f*_) values were determined. HPLC was carried out on a system consisting of two pumps, an injector and a variable UV/Vis detector, and a sodium iodide crystal detector. We have used UV absorbance 210 nm to assess the purity of the compound. HPLC was performed on a C-18 reversed phase column (C-18 Radial-Pak column, 4.6 × 150 mm, Waters, Milford, MA) with a mobile phase of water/acetonitrile, 70 : 30, using a flow rate of 0.25 mL/min. Findings were used to determine the specific activity of the final product. Tests must be completed before release of the drug product.
- *Radiochemical stability of* ^*68*^ *Ga-DOTATATE* in serum and saline was tested using HPLC and radio-TLC up to 4 hours. Test must be completed before release of the drug product.
- *Radionuclide purity*: A coaxial high purity germanium (HPGe) detector with a multichannel analyzer (MCA) (Canberra, DSA 2000, GC 8021) was used. This HPGe has an 80% relative efficiency, 1.8 keV FWHM at 1.33 MeV, and an energy range of 100 keV–10 MeV. Energy calibration was done using three NIST-traceable sources (^137^Cs (11.34 uCi 15/3/01, T_1/2_ 30.17 yrs), ^60^Co (21.34 uCi on 15/3/01, T_1/2_ 5.27 yrs), and ^133^Ba (10.13 uCi 8/1/01, T_1/2_ 3862 days)). The acceptance criteria for the radionuclide purity are that no less than 99.5% of the observed *γ*-emissions measured by HPGe should correspond to the gamma emission signature of  ^68^Ga [3].
- *Radionuclide identity*: The half-life is determined by measuring the radioactivity decay of the sample over about 100 min period. The accepted tolerance is ±5 min (^68^Ga—half-life is 68 minutes). A NIST-traceable dose calibrator Capintec Dose Calibrator (CRC-15R) with CRC-2401 Vial/Syringe Dipper (Capintec Inc., Ramsey, NJ) was used to determine the half-life.
- *Assay for radioactivity*: NIST-traceable dose calibrator (Capintec Dose Calibrator: CRC-15R) with CRC-2401 Vial/Syringe Dipper (Capintec Inc., Ramsey, NJ) was used to determine total activity (MBq or mCi) and the radioactivity concentration (MBq or mCi/mL). The dial setting for ^68^Ga provided by the vendor (dial setting = 416) was verified by a NIST traceable ^68^Ge/^68^Ge standard source (100 microCi, quoted activity uncertainty ±4%); alternatively the ^18^F dial setting can be used multiplied by (100/89) to correct for abundance difference between ^18^F (100%) and ^68^Ga (89%). A ^90^Sr constancy source (Atlantic Research Co Model B-1 S/N 252) was used to insure proper response of the Capintec dose calibrator during data collection. The ^90^Sr source was used once per month to check the well chamber response.
- *Radiochemical impurity*: No more than 5% of unbound radioactivity must be present in ^68^Ga-DOTATATE injections. Impurity level should depend on its effect on image quality and/or patient radiation absorbed dose. Test must be completed before release of the drug product.
- *Specific activity*: HPLC was used to determine specific activity. The acceptable criteria will be 0.1–1.0 Ci/*μ*mole for ^68^Ga-DOTATATE.
- *pH*: pH limits are 5.5–8.0. pH meter with pH reference standards was used. Test must be completed before release of the drug product.
- *Chemical purity*: the purity of precursor (DOTATATE) was determined by proton NMR and elemental analysis. This test is done once per batch of precursor.
- *Residual solvents*: no more than 0.04% acetonitrile and 0.5% dehydrated alcohol were to be present in the drug injection. Testing was performed using a gas chromatography system with flame ionization detection. Test must be completed before release of the drug product.
- *Bacterial endotoxins*: No more than 175/V USP EU per milliliter of the drug injection, in which V is the maximum recommended total dose, in millimeters, at the expiration time is used. Test must be completed before release of the drug product.
- *Sterility*: Sterility testing must be initiated within 24 hrs of preparation. To assure sterility, each batch of product was tested using culture vials with aerobic and anaerobic materials (NR6 and NR7, Becton Dickinson Diagnostic Instrument Systems Towson, MD). Drug solution (0.3 mL) was incubated with Bactec culture vials for 7 days at 37°C. Sterility was assayed by visualizing the cloudiness of the solution.

Additionally, manual testing was conducted to further characterize the generator to determine the following.

(i) *Elution efficiency*: The ^68^Ga elution efficiency in full 5 mL aliquots was determined using the dose calibrator and
  (1) Elution_Eff%=Ga(10 mL·aliquots)[Bq] 68Ga_loaded(decay_corrected)[Bq] 68 ×100%.
  Elution efficiency was measured for 1, 3, and 7 hr time periods in between elutes over the life cycle of the generator. Meyer et al. reported that their Russian TiO_2_ generator elution efficiency decreased from 90% at the first elution to 60% after 8 months and >500 elutes [4]. They estimated ^68^Ge loss due to breakthrough that contributed to <1% of this efficiency loss. Possibly, the decrease in elution efficiency is due to alteration in the column structure. Velikyan et al. used also the Russian TiO_2_ generator and reported similar loss of elution efficiency, down to 41% after 29-month use [5].
(ii) *Trace-metal content/purification of elution*: Trace metal ions were analyzed using an Inductively Coupled Plasma-Atomic Emission Spectra (ICP-AES) procedure. We propose obtaining this analysis once every 6 months over the project period (*n* = 3) to observe the metal content of elute as the generator ages. A daily elution of the generator is recommended in order to keep the concentration of metal ions as low as possible.

## 2. Results

**Figure 2:**
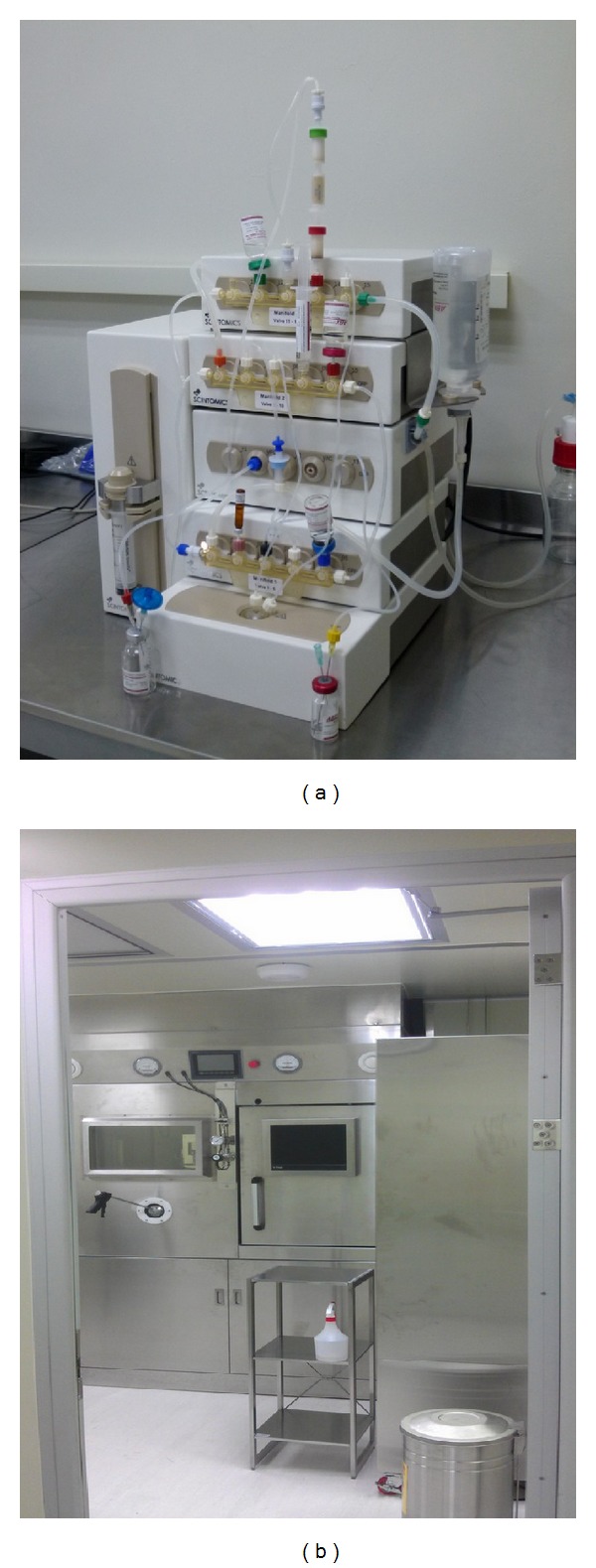
An automated synthesizer (a) is housed in a hot cell (b) for CGMP production of PET drug. Adjacent to the hot cell is the cell for dispensing of PET drug.

**Figure 3:**
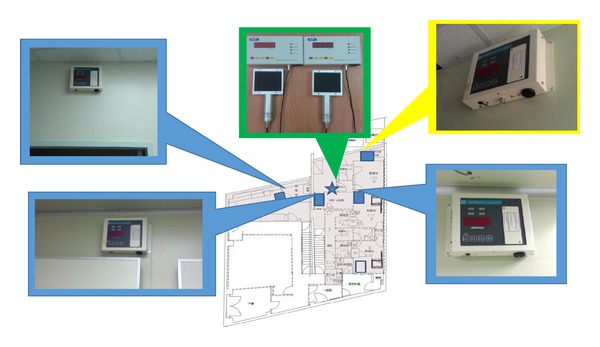
Radiation survey meters are necessary to measure radiation dose rates in the CGMP facilities.

**Figure 4:**
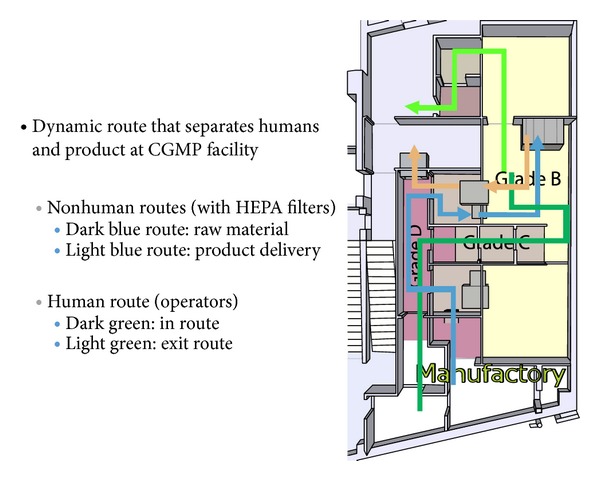
Human and nonhuman routes designs are separated in the CGMP facility.

PET Pharm Biotech firm has three connecting laboratories and a room for quality assurance. The lab has been designed and equipped to perform CGMP radiolabeling of different isotopes and molecules (Figure 1). The drug product is synthesized and dispensed in hot cells (Figure 2). Radiation survey meters are mounted in CGMP facilities to monitor radiation exposure (Figure 3). There is a system established to monitor temperature, humidity, radiation dose rate, and air pressure in CGMP facility. The ventilation control system has chambers to collect waste gas as well as to vent through hot cell. The quality assurance lab consists of Millipore water filtration system for all chemical, biological, and radiological experiments, −80°C freezer, −20°C freezer, explosion proof refrigerator, vacuum pump, analytical balance, freeze-dryer, incubator, a Gamma Counter, Capintec dose calibrator, multichannel analyzer, gas chromatography, high performance liquid chromatography (HPLC) system with UV and radioactive flow detectors, radio-TLC scanner,and Survey meter with GM detector. Our system covers the design, monitoring, and control of manufacturing processes and facilities, maintenance, calibration, and validation of equipment, condition of facilities, qualifications and training of employees, reliability and reproducibility of processes, test method validation, handling of complaints, and system inspection and auditing. The staff maintains resources to the highest technical degree. There are different routes and grades for operators and drug delivery in CGMP facility (Figure 4). A conventional PET drug production room (in grade C cleanliness) usually has multiple hot cells enclosing various synthesis modules. Each module then is connected by a dedicated tubing and through it the formulated product is pumped to a distant hot cell (grade A) located in a separate room (grade B) for aseptic filling. Here, we present a new concept for the design of a radiopharmacy and have also constructed one accordingly. With this design, we argue that multiple small grade B rooms each set up with a hot cell encasing one grade B synthesis compartment and one grade A aseptic filling compartment would be a more efficient way for complying the most current GMP, especially in a radiopharmacy where more than one kind of PET drugs is regularly produced and dispensed (Figure 1).

Our current facility fulfills PIC/s GMP Guideline and USP 〈797〉 Guideline. We have reserved sites for production of different radiopharmaceuticals. Housing an automated module in our hot cell is also compliant with cGMP and FDA 21 CFR part 11 requirements. Our system is capable of detection of radioactivity during radiopharmaceutical production. For quality control (QC) and quality assurance (QA), we have established the criteria for control of the raw materials, analytical quality control, drug product stability testing prior to clinic distribution, and data integrity. For validation process, we have established the reference standard, equipment, and facilities validation and analytical method validation. A dose calibrator and chromatographic analysis were used to assess product quality. The general acceptance criteria for PET drug product especially ^18^F-agent are shown in Table 1. For the ^68^Ga-DOTATATE labeling, the radiochemical purity of final product in our system was greater than 96%, with decay-corrected radioactive yields of 80%. The total synthesis time reported is 20 minutes, including elution time. The temperature and pressure profiles during the process were examined for consistency.

The concerned cleanroom area is including buffer, final gowning, raw materials and products in-and-out, and two production rooms. With each production room only 12 m^2^, one hot cell, and maximum two operators, grade B is easily maintained. The regular and frequent disinfection practice required for the grade B room is also achieved without any difficulties. Within hot cell, the two compartments are adjacent and, thus, the product transport line is short enough to allow the use of prepackaged disposable sterilized tubing. Importantly, two production rooms can be in operation simultaneously with the diminished risk of cross contamination. The government (Taiwan) auditors have deemed this design in compliance with PIC/S GMP requirements.

## 3. Discussion

Due to the preferential accumulation of ^18^F-FDG in tumor cells, FDG-PET can provide valuable information for tumor diagnosis. In contrast to conventional imaging modalities, FDG-PET can detect lesions with high glucose metabolism regardless of their anatomical shape or location and, therefore, can distinguish between posttherapy changes and foci of residual active disease. FDG-PET has been shown to be more accurate for the diagnosis and staging of tumors than conventional imaging methods in clinical trials [6–8]. Unfortunately, FDG has not been as advantageous for imaging neuroendocrine tumors (NETs) and only tumors with high proliferative activity and low differentiation have shown an increased FDG uptake [9].

The incidence of NETs has increased over the past several decades, due in part to improvements in discovering them at a localized stage using imaging. While the early detection of NETs allows for potentially curative treatment, the five-year survival rate for patients is less than 80% due to recurrence. The abundant expression of somatostatin receptors (sst2) is a characteristic of NETs. Several analogues are used for peptide receptor radionuclide therapy (PRRT) and ^111^In-octerotide scintigraphic imaging or PET of NET. Sensitivity of PET demonstrated its superiority over scintigraphy, MRI, and CT. The efficiency of a somatostatin analogue depends on its specific binding profile. Recently, reports have shown that ^68^Ga-DOTATOC PET improves staging of NETs by PET and target definition for fractionated stereotactic radiotherapy in patients with intracranial meningiomas [10, 11]. The affinity of ^68^Ga-DOTATATE in binding sst2 (0.2 ± 0.04 nM) has been determined to be approximately 10-fold higher than that of ^68^Ga-DOTATOC (2.5 ± 0.5 nM) [12]. Existing data show that the ^68^Ga-DOTATATE PET represents a major improvement compared to the current standard in NETs imaging. For instance, on the ^68^Ga-DOTATATE PET/CT scan, intense uptake raising suspicions for recurrent NET was observed [13]. Here, we use ^68^Ga-DOTATATE for imaging NETs due to its known affinity and exciting clinic trial results.

Cross contamination among different drug products and procedures is one of the major issues that PIC/S GMP requirements designed to avoid. Yet most PET radiopharmacies in their effort to comply with these rules still set up their production rooms with multiple hot cells so various PET drugs could be produced on the same day even simultaneously. To make the matters worse, these different products often share the same aseptic filling space. Though the production rooms are only required to maintain for grade C cleanliness, the grade A aseptic filling still needs to be stationed in a grade B background which requires a preceding grade B gowning room. The overall usage of space is not as economical as one might think and it would be hard to convince the auditors that cross contamination would be unlikely.

In summary, our one room, one product concept simplifies the work flow and allows two rooms in operation simultaneously. No different batches of PET drugs will be processed in the same hot cell space that greatly lowers the risk of cross contamination. Though now the synthesis module is set up in grade B environment, this only enhances the quality of production. In conclusion, our cleanroom design is effective and in compliance with the most current GMP.

## Figures and Tables

**Table 1:** USP and EP criteria for release of PET drug product.

PET drugs	
pH value	Endotoxin test
Radiochemistry purity	Sterility test
Radiochemistry identity	Stability test
Radionuclide purity	Residual solvents test
Radionuclide identity	Kryptofix 222 test (for FDG)
Concentration of activity	
