# Corrigendum to “Design of CGMP Production of ^18^F- and ^68^Ga-Radiopharmaceuticals”

**DOI:** 10.1155/2016/7621639

**Published:** 2016-11-09

**Authors:** Yen-Ting Chi, Pei-Chun Chu, Hao-Yu Chao, Wei-Chen Shieh, Chuck C. Chen

**Affiliations:** PET Pharm Biotech Company Limited, 1-2F No. 143 and 1-2F No. 141 HouGang, Xinzhuang District, New Taipei City 24257, Taiwan

The published version of the article titled “Design of CGMP Production of ^18^F- and ^68^Ga-Radiopharmaceuticals” [1] failed to indicate that the “automated synthesizer” pictured in Figure 2(a) of the article was designed and developed by Scintomics GmbH. The synthesizer was purchased by PET Pharm Biotech Company Limited from Scintomics GmbH and installed by engineers employed by Scintomics GmbH. The authors wish to make it clear that they had no role in the development of the software or hardware of the synthesizer nor in its installation in the hot cell, as described in Sections 1.6 and 1.7 of the paper. We apologize to Scintomics GmbH and our readers for the potentially misleading oversight.

## References

[1] Chi Y.-T., Chu P.-C., Chao H.-Y., Shieh W.-C., Chen C. C. (2014). Design of CGMP production of ^18^F- and ^68^Ga-radiopharmaceuticals. *BioMed Research International*.

